# Supplementary material for: Effect of Drying Methods on Volatile Compounds of Citrus reticulata Ponkan and Chachi Peels as Characterized by GC-MS and GC-IMS
Source: Foods. 2022 Sep 1;11(17):2662. doi: 10.3390/foods11172662 (PMC9455753; doi:10.3390/foods11172662)
Supplement: Supplementary file 1 [file foods-11-02662-s001.zip › foods-1868072-supplementary.pdf]

Supplementary Material

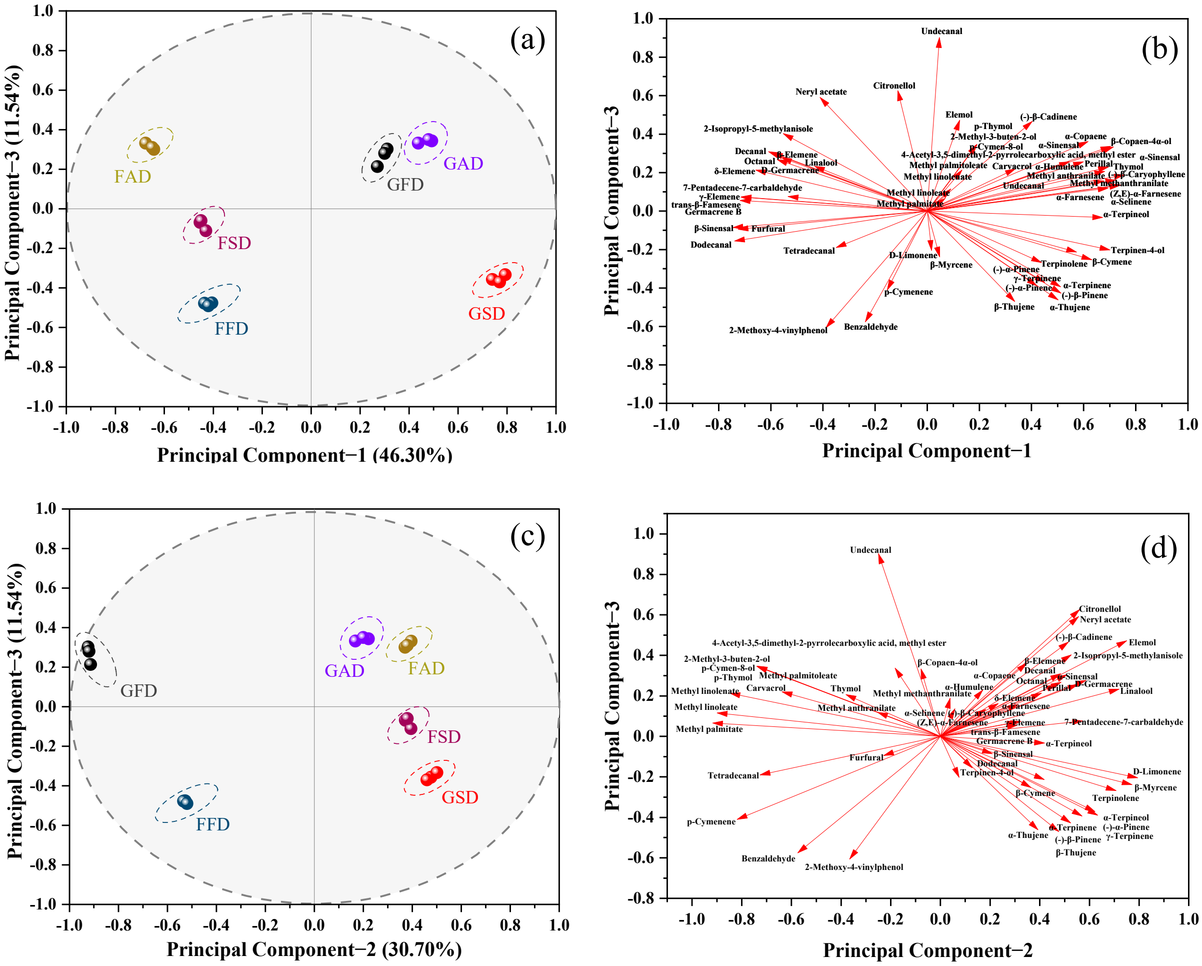

Figure S1. Plots of principal component scores (a, principal component−1 vs. principal component−3; c, principal component−2 vs. principal component−3) and loadings (b, principal component−1 vs. principal component−3; d, principal component−2 vs. principal component−3) in PCA analysis for GC-MS data of dried Chachi and Ponkan peels prepared from three drying methods.

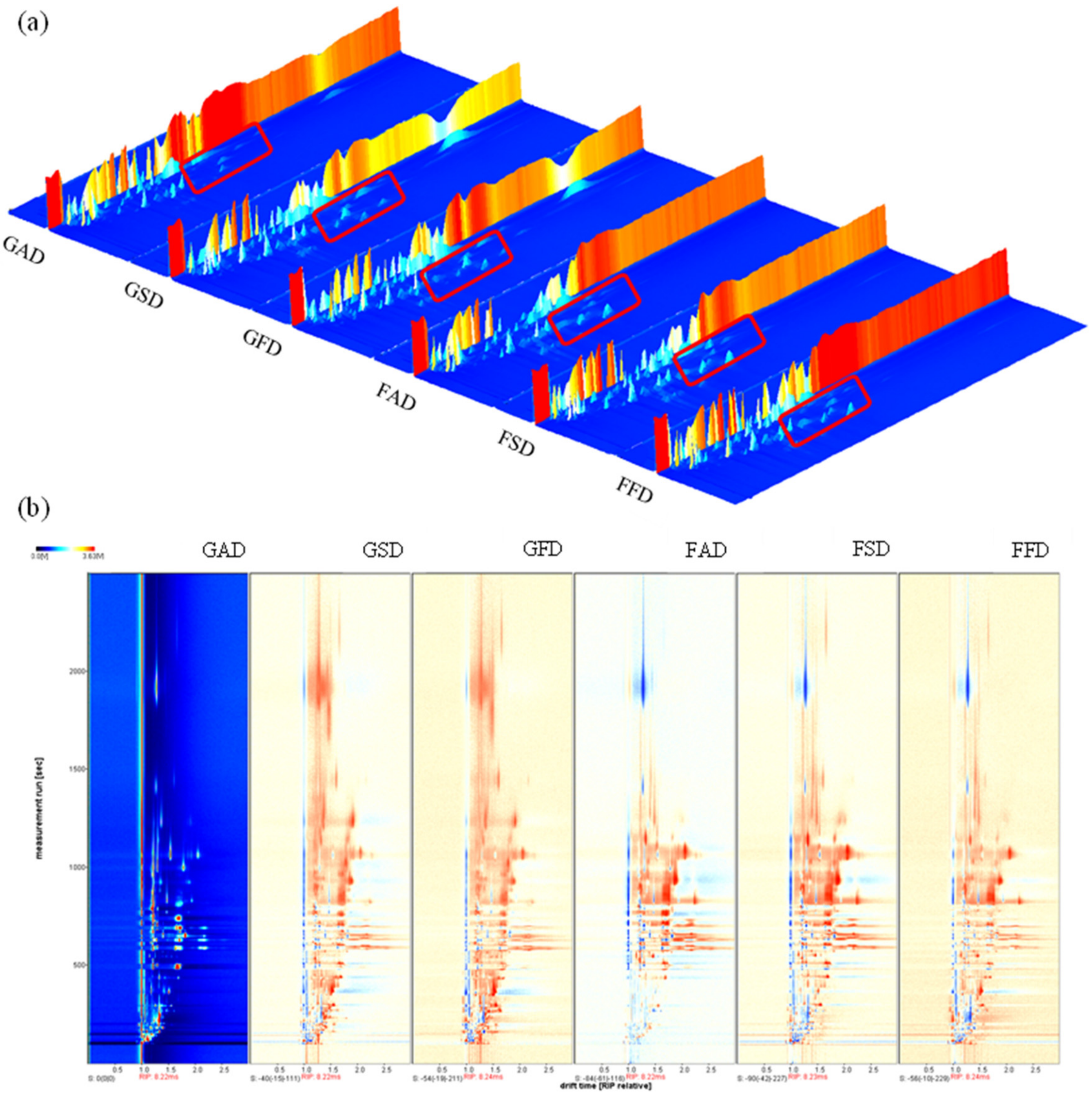

Figure S2. 3D-topographic view (a) and vertical view (b) of volatile compounds in dried Chachi and Ponkan peels prepared from three drying methods. ((a), X-, Y-, and Z-axes represent the ion migration time, retention time of GC, and ion peak strength, respectively). (b), GAD was used as a reference. The colors indicate the signal strengths of the individual compounds. Red means high intensity, and blue means low intensity.) of volatile compounds in dried Chachi and Ponkan peels prepared from three drying methods.)

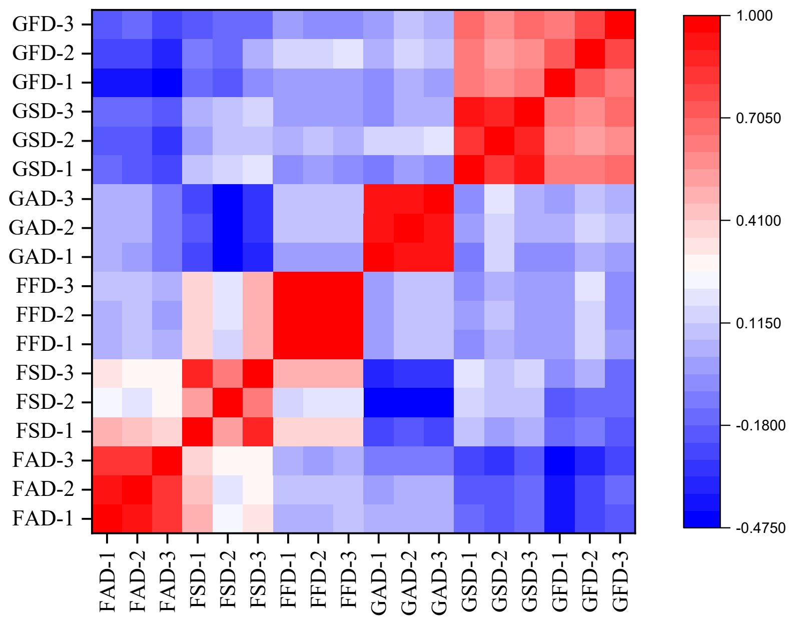

Figure S3. Correlation between dried citrus peel samples of different groups (FAD, FSD, FFD, GAD, GSD, GFD) as characterized by GC-IMS analysis.

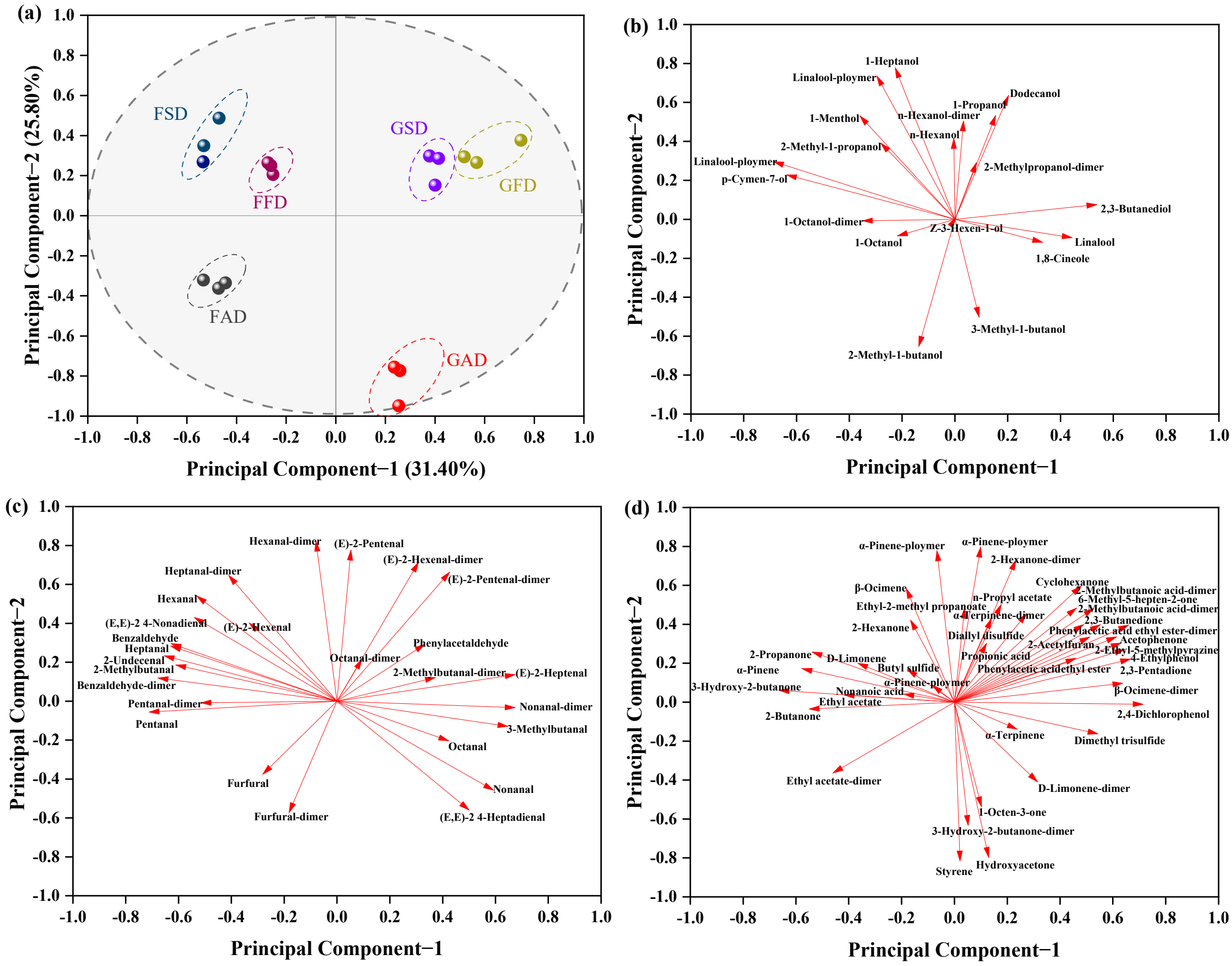

Figure S4. Plots of principal component scores (a) and loadings (b, c, d) in PCA analysis for GC-IMS data of dried Chachi and Ponkan peels prepared from three drying methods.

Table S1. The relative percentage content of each compound in GC–MS total ion chromatogram of different dried citrus peel samples through peak area normalization.

**Compound**	**CAS**	**Formula**	**FAD**	**FSD**	**FDD**	**GAD**	**GSD**	**GFD**
Alcohols								
2-Methyl-3-buten-2-ol	115-18-4	$C_5H_{10}O$	nd	nd	nd	nd	nd	0.30±0.01
Linalool	78-70-6	$C_{10}H_{18}O$	3.84±0.04^a	2.95±0.05^b	1.53±0.03^c	0.93±0.00^d	0.61±0.00^e	0.27±0.00^f
Terpinen-4-ol	562-74-3	$C_{10}H_{18}O$	0.43±0.00^d	0.38±0.00^d	1.17±0.07^a	0.45±0.01^d	0.67±0.04^c	0.93±0.01^b
p-Cymen-8-ol	1197-01-9	$C_{10}H_{14}O$	nd	nd	nd	nd	nd	0.29±0.00
α-Terpineol	98-55-5	$C_{10}H_{18}O$	2.54±0.04^b	1.96±0.04^c	4.44±0.18^a	1.95±0.01^c	1.96±0.03^c	2.66±0.03^b
Citronellol	106-22-9	$C_{10}H_{20}O$	0.59±0.02^a	0.26±0.02^b	nd	0.17±0.03^c	0.13±0.00^c	0.25±0.04^b
Elemol	639-99-6	$C_{15}H_{26}O$	0.53±0.01^a	0.21±0.02^c	nd	0.37±0.02^b	0.15±0.00^d	nd
β-Copaen-4α-ol	124753-76-0	$C_{15}H_{24}O$	nd	nd	nd	0.54±0.00^b	0.28±0.03^c	0.63±0.01^a
		**Sum**	**7.93±0.11^a**	**5.77±0.06^c**	**7.14±0.29^b**	**4.52±0.01^e**	**3.89±0.10^f**	**5.33±0.06^d**
Aromatic hydrocarbons and ethers								
β-Cymene	535-77-3	$C_{10}H_{14}$	0.31±0.00^c	0.28±0.00^c	0.55±0.10^b	0.41±0.01^c	0.88±0.01^a	0.56±0.08^b
p-Cymenene	1195-32-0	$C_{10}H_{12}$	nd	nd	2.19±0.21^a	nd	nd	0.51±0.02^b
2-Isopropyl-5-methylanisole	1076-56-8	$C_{11}H_{16}O$	0.93±0.03^a	0.52±0.01^b	nd	0.18±0.01^c	nd	nd
		**Sum**	**1.24±0.03^b**	**0.81±0.00^{cd}**	**2.75±0.31^a**	**0.59±0.01^d**	**0.88±0.01^{cd}**	**1.07±0.09^{bc}**
Phenols								
p-Thymol	3228-02-2	$C_{10}H_{14}O$	nd	nd	nd	nd	nd	0.44±0.01
Thymol	89-83-8	$C_{10}H_{14}O$	0.63±0.01^{cd}	0.47±0.05^d	1.83±0.17^a	0.84±0.00^b	0.68±0.01^{bc}	1.86±0.02^a
Carvacrol	499-75-2	$C_{10}H_{14}O$	nd	nd	nd	nd	0.14±0.02^b	1.10±0.00^a
2-Methoxy-4-vinylphenol	7786-61-0	$C_9H_{10}O_2$	0.84±0.02^b	0.46±0.02^c	8.20±0.34^a	0.15±0.00^c	0.13±0.03^c	0.30±0.02^c
		**Sum**	**1.47±0.01^c**	**0.92±0.07^d**	**10.03±0.51^a**	**1.00±0.00^{cd}**	**0.95±0.01^{cd}**	**3.70±0.00^b**
N-containing compounds								
Methyl anthranilate	134-20-3	$C_8H_9NO_2$	nd	nd	nd	0.15±0.01^c	0.20±0.00^b	0.48±0.03^a
Methyl methanthranilate	85-91-6	$C_9H_{11}NO_2$	5.47±0.63^e	1.92±0.13^f	10.87±0.78^d	42.31±0.37^b	30.71±0.39^c	47.52±0.09^a
4-Acetyl-3,5-dimethyl-2-pyrrole carboxylic acid, methyl ester	89909-47-7	$C_{10}H_{13}NO_3$	nd	nd	nd	0.16±0.04^b	0.09±0.00^c	0.24±0.01^a
		**Sum**	**5.47±0.63^e**	**1.92±0.13^f**	**10.87±0.78^d**	**42.61±0.32^b**	**31.00±0.39^c**	**48.23±0.07^a**
Aldehydes								
Furfural	98-01-1	$C_5H_4O_2$	1.57±0.22^b	0.91±0.00^c	4.01±0.17^a	0.35±0.03^d	0.12±0.00^d	0.80±0.05^c
Benzaldehyde	100-52-7	C_7H_6O	nd	nd	1.29±0.01^a	nd	nd	0.18±0.05^b
Octanal	124-13-0	$C_8H_{16}O$	0.28±0.00^a	0.19±0.00^b	nd	nd	nd	nd

Decanal	112-31-2	$C_{10}H_{20}O$	1.77±0.00^{b}	1.12±0.02^{c}	1.92±0.07^{a}	0.48±0.01^{e}	0.28±0.00^{f}	0.62±0.06^{d}
Perillal	2111-75-3	$C_{10}H_{14}O$	0.68±0.01^{b}	0.53±0.01^{d}	nd	0.76±0.01^{a}	0.62±0.01^{c}	0.60±0.00^{c}
Undecanal	112-44-7	$C_{11}H_{22}O$	0.24±0.01	nd	nd	0.13±0.00^{b}	nd	0.23±0.01^{a}
Dodecanal	112-54-9	$C_{12}H_{24}O$	1.81±0.01^{b}	0.83±0.04^{c}	3.46±0.14^{a}	nd	nd	nd
Tetradecanal	124-25-4	$C_{14}H_{28}O$	0.26±0.03^{b}	nd	1.21±0.10^{a}	nd	nd	0.31±0.00^{b}
β-Sinensal	60066-88-8	$C_{15}H_{22}O$	3.08±0.20^{b}	1.60±0.00^{c}	4.87±0.19^{a}	nd	nd	nd
α-Sinensal	17909-77-2	$C_{15}H_{22}O$	4.01±0.32^{b}	2.37±0.01^{c}	3.56±0.36^{b}	7.62±0.33^{a}	3.90±0.25^{b}	2.40±0.17^{c}
7-Pentadecene-7-carbaldehyde	——	$C_{16}H_{30}O$	1.45±0.09^{a}	1.29±0.02^{b}	0.59±0.03^{c}	0.14±0.04^{d}	0.15±0.01^{d}	nd
		**Sum**	**15.16±0.84^{b}**	**8.83±0.05^{c}**	**20.91±0.44^{a}**	**9.48±0.32^{c}**	**5.07±0.27^{d}**	**5.14±0.02^{d}**
Monoterpenes								
α-Thujene	2867-05-2	$C_{10}H_{16}$	nd	nd	nd	nd	0.27±0.00	nd
(-)-α-Pinene	7785-26-4	$C_{10}H_{16}$	0.29±0.00^{c}	0.62±0.01^{b}	nd	0.23±0.00^{d}	0.86±0.03^{a}	nd
β-Thujene	28634-89-1	$C_{10}H_{16}$	nd	0.16±0.01^{a}	nd	nd	0.10±0.00^{b}	nd
(-)-β-Pinene	18172-67-3	$C_{10}H_{16}$	nd	0.30±0.01^{b}	nd	0.17±0.01^{c}	0.65±0.02^{a}	nd
β-Myrcene	123-35-3	$C_{10}H_{16}$	0.91±0.00^{b}	1.62±0.02^{a}	nd	0.27±0.01^{d}	0.79±0.02^{c}	nd
α-Terpinene	99-86-5	$C_{10}H_{16}$	nd	0.27±0.01^{b}	nd	0.17±0.00^{c}	0.43±0.00^{a}	nd
D-limonene	5989-27-5	$C_{10}H_{16}$	39.87±0.32^{b}	60.73±0.01^{a}	6.89±0.83^{e}	12.17±0.28^{d}	28.76±0.58^{c}	2.02±0.67^{f}
γ-Terpinene	99-85-4	$C_{10}H_{16}$	3.97±0.02^{c}	6.45±0.00^{b}	1.09±0.10^{e}	3.25±0.09^{d}	10.09±0.14^{a}	0.55±0.14^{f}
Terpinolene	586-62-9	$C_{10}H_{16}$	0.53±0.00^{c}	0.57±0.01^{b}	nd	0.41±0.00^{d}	0.85±0.02^{a}	nd
		**Sum**	**45.57±0.35^{b}**	**70.72±0.02^{a}**	**7.98±0.93^{e}**	**16.66±0.40^{d}**	**42.80±0.77^{c}**	**2.56±0.81^{f}**
Sesquiterpenes								
δ-Elemene	20307-84-0	$C_{15}H_{24}$	3.66±0.01^{a}	1.76±0.03^{b}	1.89±0.18^{b}	0.11±0.02^{c}	nd	nd
α-Copaene	3856-25-5	$C_{15}H_{24}$	0.50±0.00^{b}	0.21±0.00^{d}	0.51±0.04^{b}	0.78±0.02^{a}	0.43±0.01^{c}	0.50±0.00^{b}
β-Elemene	515-13-9	$C_{15}H_{24}$	2.12±0.02^{a}	1.03±0.03^{c}	1.52±0.03^{b}	0.37±0.00^{d}	0.18±0.00^{f}	0.27±0.00^{e}
(-)-β-Caryophyllene	87-44-5	$C_{15}H_{24}$	0.50±0.07^{c}	0.20±0.02^{d}	0.52±0.03^{c}	2.81±0.04^{a}	1.95±0.08^{b}	1.92±0.02^{b}
γ-Elemene	29873-99-2	$C_{15}H_{24}$	1.77±0.00^{a}	0.89±0.01^{b}	1.71±0.15^{a}	nd	nd	nd
trans-β-Famesene	18794-84-8	$C_{15}H_{24}$	2.34±0.01^{a}	1.09±0.03^{b}	2.33±0.11^{a}	nd	nd	nd
α-Humulene	6753-98-6	$C_{15}H_{24}$	nd	nd	nd	0.49±0.02^{a}	0.26±0.01^{b}	0.28±0.01^{b}
D-Germacrene	23986-74-5	$C_{15}H_{24}$	3.24±0.04^{a}	1.50±0.01^{c}	2.08±0.18^{b}	0.62±0.02^{d}	0.25±0.01^{e}	nd
(Z,E)-α-Farnesene	26560-14-5	$C_{15}H_{24}$	nd	nd	nd	0.46±0.00^{a}	0.32±0.01^{b}	0.33±0.00^{b}
α-Selinene	473-13-2	$C_{15}H_{24}$	nd	nd	0.88±0.05^{d}	1.75±0.04^{a}	1.13±0.03^{c}	1.51±0.02^{b}
α-Farnesene	502-61-4	$C_{15}H_{24}$	3.23±0.05^{d}	1.50±0.02^{e}	3.17±0.23^{d}	16.10±0.26^{a}	10.02±0.37^{b}	7.19±0.18^{c}
(-)-β-Cadinene	523-47-7	$C_{15}H_{24}$	2.01±0.04^{b}	1.00±0.00^{e}	2.72±0.05^{a}	1.49±0.01^{c}	0.86±0.03^{f}	1.27±0.02^{d}
Germacrene B	15423-57-1	$C_{15}H_{24}$	3.33±0.03^{a}	1.66±0.00^{b}	3.44±0.62^{a}	nd	nd	nd

		**Sum**	**22.71±0.01^{b}**	**10.84±0.12^{f}**	**20.77±1.58^{c}**	**25.00±0.40^{a}**	**15.41±0.56^{d}**	**13.28±0.25^{e}**
Esters								
Neryl acetate	141-12-8	$C_{12}H_{20}O_2$	0.46±0.01^{a}	0.20±0.01^{b}	nd	0.14±0.00^{c}	nd	nd
Methyl palmitoleate	1120-25-8	$C_{17}H_{32}O_2$	nd	nd	nd	nd	nd	0.46±0.02
Methyl palmitate	112-39-0	$C_{17}H_{34}O_2$	nd	nd	12.15±0.16^{a}	nd	nd	10.72±0.31^{b}
Methyl linoleate	112-63-0	$C_{19}H_{34}O_2$	nd	nd	5.21±0.43^{a}	nd	nd	5.59±0.03^{a}
Methyl linolenate	301-00-8	$C_{19}H_{32}O_2$	nd	nd	2.19±0.22^{b}	nd	nd	3.92±0.15^{a}
		**Sum**	**0.46±0.01^{c}**	**0.20±0.01^{c}**	**19.55±0.80^{b}**	**0.14±0.00^{c}**	**nd**	**20.69±0.52^{a}**
		**Total**	**100.00%**	**100.00%**	**100.00%**	**100.00%**	**100.00%**	**100.00%**

The term "nd" means the compound was not detected in sample. Different lower-case letters in the same row indicated the significant differences ($P < 0.05$).
